# 

**Published:** 1889-08-03

**Authors:** 


### SPECIAL ANNOUNCEMENT.

### MISS FLORENCE NIGHTINGALE.

The next (September) monthly part of The Hospital will be accompanied by a portrait of Miss Nightingale, which has been reproduced at great cost and perfection; and it will at last be possible for every member of the nursing profession to possess the likeness of one who, by a life of self-sacrifice and philanthropic labour, has raised herself to pre-eminence among women. The monthly number will be issued at sixpence as usual, but orders should be sent early.

We regret that, owing to an accident which has happened to the plate at the collotyping works where this portrait is being produced, we were compelled to postpone its publication to the next (September) monthly part. Though the delay thus caused is much to be regretted, there is consolation in the knowledge that a really excellent portrait of Miss Nightingale will be within reach of our readers a few weeks hence. The biographical sketch appeared in our issue of July 27.

### NOTICE TO CORRESPONDENTS.

All MS., Letters, Books for Review, and other matters intended for the Editor, should be addressed The Editor, The Lodge, Porchester Square, London, W.

### Notice to Advertisers and Subscribers.

All Advertisements, Orders for Copies of The Hospital, and Business Communications should be addressed to The Publisher, *not to the Editor*, The Hospital, 139-140, Salisbury-court, Fleet-street, E.C.

Vol. V., now ready, 4s., post free. Cases for binding, may be had of the Publisher, at 1s. 6d. each.

### To Residents, Secretaries, and Matrons.

The Editor will be glad to have the Regulations and each Report as issued by Asylums, Hospitals, Convalescent and Nursing Institutions, as well as those of other Charities, and an early copy in duplicate of all Appeals and Festival Notices, etc., addressed to *The Editor, The Lodge, Porchester-square, London, W.* Any superintendent, secretary, matron, or other chief official who regularly sends such information may be registered, on application to the Publisher, to receive free of cost a cover or binding The Hospital in half-yearly volumes.

### Amusements and Relaxation.

### Second Quarterly Word Competition Commenced July 6th, ends September 28th.

Three Prizes of 15s., 10s., 5s., will be given for the largest number of words derived from the words set for dissection.

Proper names, abbreviations, foreign words, words of less than four letters, and repetitions are barred; plurals, and past and present participles of verbs, are allowed. Nuttall's *Standard* dictionary only to be used.

N.B.—Word dissections must be sent in WEEKLY, arranged alphabetically, with correct total affixed.

The word for dissection for this, the *Fifth* week of the quarter, being

"OSBORNE."

**Results of Third Week.**

| Names. | July 25th. | Totals. | Names. | July 25th. | Totals. |
|---|---|---|---|---|---|
| Nurse Duty | 47 | 237 | Rover | — | 165 |
| N'importe Qui | 45 | 209 | Leirion | 46 | 176 |
| Jumps | — | 30 | Matron | 45 | 132 |
| Tinie | — | 186 | F. C. J. | 43 | 163 |
| Patience | 46 | 227 | Percy Verance | — | 18 |
| Amicus | 42 | 225 | Gleniffer | 46 | 175 |
| Broxbourne | 47 | 230 | Gypsye | 40 | 197 |
| Speedwell | 46 | 216 | Ladyr | 27 | 127 |
| Sister | 47 | 221 | A. B. | 41 | 170 |
| Lightowlers | 47 | 230 | Essie | 45 | 178 |
| Lauriston | 47 | 227 | W. G. R. | 37 | 180 |
| Rattler | — | 28 | Cowboy Bill | 46 | 179 |
| Paignton | 47 | 179 | M. L. A. | 30 | 117 |
| Fassifern | — | 141 | Little Tuppy | 33 | 110 |
| W. R. E. | 48 | 227 | Embryo | — | 56 |
| Esperance | 49 | 229 | K. Jarman | — | 43 |
| M. W. | 48 | 234 | Reveal | 47 | 196 |
| Isabel | 47 | 215 | Jenny Wren | 32 | 163 |
| Buxton | 28 | 150 | Condorcet | 38 | 194 |
| Nux Vom | 46 | 213 | Electrician | 27 | 27 |
| Qu'appelle | 48 | 206 | Pearl | 38 | 38 |
| Spes | — | 24 | R. W. | 43 | 43 |
| Secnarf | 47 | 212 | Nurse Amie | — | 89 |

**Notice to Correspondents.**

N.B.—All letters referring to this page which do not arrive at 139 and 140, Salisbury-court, London, E.C., by the *first post on Thursdays*, and are not addressed PRIZE EDITOR, will in future be disqualified and disregarded

Competitors can enter for *all* quarterly competitions, but no competitor may take more than *two quarterly prizes* during the year.

### Scraps and Gleanings.

There is an outbreak of measles at Coventry.

A hospital for birds is the latest institution opened in Chicago.

Devonport Hospital Saturday Fund this year nearly reaches £100.

William Brixton died last week at St. Mary's Hospital from hydrophobia.

Canon Farrar preached last Sunday in aid of the seaside camp for London boys.

The mortality amongst illegitimate children boarded out in France is from 35 to 40 per cent.

The Prince of Wales dined with the Stewards of St. Bartholomew's Hospital on July 24th.

The Earl Percy will open the Victoria Jubilee Infirmary, at Tynemouth, on August 3rd.

On August 12th an examination will be held for eight commissions in the army medical staff.

The City Corporation have voted fifty guineas to the Children's Country Holiday Fund.

Mr. William Anderson will deliver the opening address at St. Thomas's on October 1st.

Dr. Cyril Jecks has been appointed resident medical officer to University College Hospital.

Cardinal Manning lately opened St. Veronica's Retreat in Chiswick, a home for female inebriates.

It is proposed to celebrate the Queen's visit to Wales by the erection of a hospital to bear her name.

Dr. Sidney Coupland is preparing for the press the last work of the late Wilson Fox—"Diseases of the Lungs."

Murray's Magazine for August contains an article by Mr. William Covington—"Is Hospital Sunday a Failure?"

The *tableaux vivants* at Shrewsbury, to raise funds to supply new beds to Salop Infirmary, were a great success.

The Duchess of Albany visited the Princess Alice Memorial Hospital on July 19th, and took tea in the matron's room.

The Marchioness of Dufferin will publish a selection from her Indian journals under the title of "Our Viceregal Life in India."

Mr. Van Praagh has been presented with a silver salver by the committee of the Central London Throat and Ear Hospital.

The subscription list for Halifax New Infirmary now reaches £49,426. The matron of the Borough Hospital has given two guineas.

A bazaar was held at Knightsbridge last Saturday in aid of Lady Sandhurst's Cripples Home, and the Hutton Home, Sunbury.

Sir Frederick Abel, the well-known chemist, will be the president of the meeting of the British Association next year at Leeds.

The other day a cricket match between the doctors and lawyers was played at Sunderland, and the proceeds given to the Infirmary.

The Queen proposes, it is said, to visit Strathpeffer in the autumn, for the purpose of taking a course of the baths for which that place is famous.

A fatal case of "local" cholera is reported from a place near Buda-pest, and four relatives of the deceased are said to be suffering from the malady.

Surgeon Keatinge is in charge of a floating hospital, consisting of five dahabiehs, which has gone up the Nile towards the seat of the present skirmishes.

The bazaar recently held by Mrs. Arfwedson was the means of benefiting the funds of the Samaritan Hospital to the extent of £80, that amount being the net proceeds.

At Corbeil a cat went mad and bit several other tabbies, then the villagers decreed and executed the death of all the cats in the neighbourhood. Oh, to be a mouse at Corbeil!

Mrs. Peel's "At Home," in aid of the Children's Country Holidays Fund, was very successful. She has been able to transmit to the treasurer, the Hon Alfred Lyttelton, over £160.

The Rev. Astley Cooper has sent the sum of £14 13s. 5d. to Mrs. Jeune towards her fund for giving poor and sickly London children a holiday in the country. The amount was collected in the Hickey's Almshouse.

The Lord Mayor has received from the Delegate Chief Rabbi (Rev. Dr. H. Adler) a cheque for £657 15s. 8d., being the amount subscribed by the various Jewish congregations in London for the Hospital Sunday Fund.

The Medical Officer of Health for Kensington, in his latest report, writes feelingly of the nuisance from brick-burning in the outlying parts of London. The odour from the brickfields is insupportable, especially at night.

At the Wicklow Assizes, Mrs. Bright, until recently matron of Armagh Lunatic Asylum, was awarded £700 damages against the Northern Railway Company for injuries received in the Armagh railway disaster.

Dr. Olivier, of Havre, advises people to be careful in drinking cider. It is made, as a rule, with stagnant water, the microbes in which do not perish during fermentation. On the contrary, the fever germs thrive upon the juice of the apple.

Dr. Anna Bayer, of Berne, has received a memorial from thirty Women's Societies in Bohemia and Moldavia, begging her to devote her talents to her native country. The Austrian law, however, forbids a woman to practice medicine.

The committee of "The Gordon Boy's Home" made their annual inspection of the home and its inmates on August 1, when the replica of the picture of General Gordon, entitled, "The Last Watch," was presented by the artist, Mr. Lowes Dickenson, to the institution.

At St. Michael's, Folkestone, the other day, the Rev. E. Husband, in announcing a "flower service" in the afternoon for the benefit of the workhouse, invited his congregation to bring, not only flowers and fruit for the children, but also offerings of tea for the old women, and tobacco for the old men.

